# A case report of human tularemia from Iran

**Published:** 2018-08

**Authors:** Mahdi Rohani, Behzad Mohsenpour, Ahmad Ghasemi, Saber Esmaeili, Mohammad Karimi, Heinrich Neubauer, Herbert Tomaso, Ehsan Mostafavi

**Affiliations:** 1 Department of Microbiology, Pasteur Institute of Iran, Tehran, Iran; 2 National Reference Laboratory for Plague, Tularemia and Q Fever, Research Centre for Emerging and Reemerging Infectious Diseases, Pasteur Institute of Iran, Akanlu, Kabudar Ahang, Hamadan, Iran; 3 Zoonosis Research Center, Kurdistan University of Medical Sciences, Sanandaj, Iran; 4 Department of Bacteriology, Faculty of Medical Sciences, Tarbiat Modares University, Tehran, Iran; 5 Centre for Communicable Diseases Control, Deputy of Health, Kurdistan University of Medical Sciences, Sanandaj, Iran; 6 Federal Research Institute for Animal Health, Friedrich-Loeffler-Institut, Jena, Germany; 7 Department of Epidemiology and Biostatistics, Research Centre for Emerging and Reemerging Infectious Diseases, Pasteur Institute of Iran, Tehran, Iran

**Keywords:** *Francisella tularensis*, Hare, Tularemia, Iran

## Abstract

Tularemia is one of the most contagious bacterial infections. Here, we report a human case of glandular tularemia in Iran following the first report in 1980. The patient was a 6-year-old girl who had consumed a hunted hare in Kurdistan Province in western Iran.

## INTRODUCTION

*Francisella tularensis* is a Gram-negative bacterium and the causative agent of tularemia, which is regarded as one of the most important zoonotic bacterial diseases worldwide (1). This agent can infect more than 200 species of vertebrates and may be transmitted to humans by ingestion of contaminated water or food, handling infected animals, arthropod bites, or inhalation of contaminated aerosols (2). Farmers, veterinarians, hunters, and medical health workers are among the people at greatest risk of infection (1). Tularemia is widely spread throughout the northern hemisphere. Some of the neighboring countries of Iran such as Turkey, Kazakhstan, Turkmenistan and Russia report tularemia cases annually (3–5).

In 1969 and 1970, tularemia was reported in domestic and wild animals from the northwest (Azerbaijan province) and southeast (Zabol County) of Iran (6). The first and only human case of tularemia in Iran was reported in 1980 in Marivan County, Kurdistan Province (7). Following the establishment of the National Reference Laboratory of Plague and Tularemia in 2011 in the Pasteur Institute of Iran, tularemia surveillance was increased throughout the country. Serological surveys among high-risk human populations demonstrated 14.4% and 6.5% seroprevalence of the disease in the west (Kurdistan Province, 2011) and east of Iran (Sistan and Baluchestan Province, 2012), respectively (8, 9). Seropositive rodents were also reported in two studies from Kurdistan (2013) and Sistan and Baluchestan (2013) provinces (10). Due to the emerging prevalence of *F. tularensis* in Kurdistan Province, educational programs were initiated to increase awareness of physicians for the disease and to consider tularemia in their differential diagnosis.

This paper reports *F. tularensis* infection in a patient in Kurdistan Province following the consumption of meat of a hunted hare in 2017.

### Case study

In February 2017 a 6-year-old girl from a village near Marivan City in Kurdistan Province was admitted to a hospital in Sanandaj County, Kurdistan Province. Four days after consuming the meat of a hunted hare, rashes appeared on her body, forearm, and legs; however, 2 hrs later, these rashes disappeared without leaving any lesions or scars. At time of admission, she had no signs of fever, nausea, vomiting, or a sore throat. She had mild lymph-adenopathy of the axillary lymph nodes. Based on the epidemiological history (consuming meat of a hunted hare and living within an area with previous reports of tularemia) and clinical signs (lymph-adenopathy and rash), the patient was hospitalized, considered as a suspected case of tularemia and gentamicin was administrated after taking a blood sample.

Sonographic examination revealed that the liver was larger than normal (130 mm in the Medio clavicular line). Kidneys (78 mm in length), spleen, and gallbladder were normal. The blood culture of the hospital laboratory was negative after 48 h of incubation. Urine culture was also negative. The sample was sent to the Pasteur Institute of Iran for molecular and serological detection. In the biochemical examination, the concentration of BS, K^+^, and BUN was normal but Cr (0.5) and Na^+^(132) values were lower than normal. ESR, WBC, Hb, and PTT were normal but CRP was positive (1+).

**Fig. 1.**
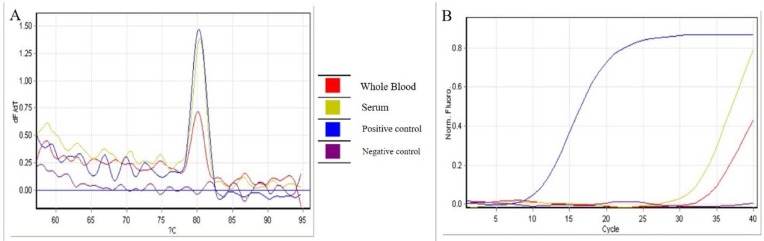
The amplification plots of the ISFtu2 real-time PCR assay. A: melting curve of ISFtu2 PCR. B: Quantification plots of ISFtu2 PCR.

Serology and molecular detection were performed in the reference laboratory for tularemia. In the first step, the rapid test VIRAPID^®^ TULAREMIA strip (Vircell, Spain) was used and then the IgM and IgG reactions were quantified in two paired samples taken with 21-day intervals with ELISA (Virion-Serion, Germany). According to the WHO guideline for tularemia (11), a single serum/plasma specimen with a positive ELISA titer is presumptive for *F. tularensis*. In this case, IgM titer (24 and 21 U/ml, respectively) was positive in both samples. For molecular detection, DNA was extracted from a blood sample using the QIAamp DNA Mini Kit (Qiagen, Germany) and the ISFtu2 real-time PCR was applied. This gene is present in the *F. tularensis* genome in various copy numbers (12) and the assay; therefore, is suitable for detection of the presence of this bacterium in blood samples. After positive results, the presence of the *fop-A* gene was examined to confirm the presence of *F. tularemia* (Fig. 1). The sequence of primers and probes, described previously, were ISFtu2F, TTGGTAGATCAGTTGGTAGGATAACC, ISFtu2R TGAGTTTTATCCTCTGACAACAATATTTC, ISFtu2P FAM-AAAATCCATGCTATGACTGATGCTTTAGGTAATCCA-TAMRA and *fopA*-F AACAATGGCACCTAGTAATATTTCTGG, *fopA*-R CCACCAAAGAACCATGTTAAACC, *fopA*P FAM-TGGCAGAGCGGGTACTAACATGATTGGT-TAMRA by amplification sizes of 97 and 87 bp, respectively. The DNA of *F. tularensis* subsp *holarctica* NCTC 10857 was used as a positive control. The amplified product of the ISFtu2 gene was sequenced and compared with the GenBank database. After 2 days of gentamicin administration, the child left the hospital with a good general condition and alleviated symptoms.

## CONCLUSION

This report of tularemia from the Kurdistan Province was the first human case of tularemia since 1980 from this region (7). Recent serological surveys of human and animal populations have indicated presence of tularemia in this area (9). Some further studies on environmental water samples of the region have also shown that *F. tularensis* is endemic in this province (data not published). Lack of tularemia reports over a period of 37 years can be due to an insufficient systematic surveillance system to monitor the bacteria in humans, animals, and the environment. According to our report, it seems that the healthcare guidelines for tularemia should be revised and physicians and laboratory staff must be made aware of the circulation of this agent in the area. For this purpose, some rapid tests can be made available for local and regional laboratories as well.

Further work is needed to identify current reservoirs, modes of transmission, and the biotype of *F. tularensis* circulating in this region. Moreover, it is expected that more human cases in Kurdistan Province will be found soon by implementing more educational programs for all physicians and healthcare workers to improve their awareness level about the disease.
